# The effectiveness of metabolic resistance training versus traditional cardio on athletic performance: a systematic review and meta-analysis

**DOI:** 10.3389/fphys.2025.1551645

**Published:** 2025-03-20

**Authors:** Yu Tongwu, Ding Chuanwei

**Affiliations:** ^1^ Capital University of Physical Education And Sports, Beijing, China; ^2^ Anhui Communications Vocational & Technical College, Hefei, China

**Keywords:** metabolic resistance training (MRT), high-intensity interval training (HIIT), athletic performance, athlete development, meta-analysis

## Abstract

**Introduction:**

The “no pain, no gain” philosophy has long influenced athletic training approaches, particularly in high-intensity workouts like metabolic resistance training (MRT). However, the necessity of discomfort-inducing training for optimal athletic performance remains debatable. This systematic review and meta-analysis examined whether MRT provided comparable or better results than traditional training methods in trained athletes.

**Methods:**

A systematic search of PubMed/MEDLINE, Web of Science, Scopus, and SPORTDiscus (January 2004 - December 2024) identified RCTs comparing MRT with traditional training in athletes. Two reviewers screened studies and assessed bias risk using Cochrane RoB 2. Random - effects meta - analyses were conducted for outcomes like VO2max, peak power, sprint performance, blood lactate, time to exhaustion, and jump height. GRADE was used to evaluate evidence certainty.

**Results:**

Eleven studies (n = 276 participants) met inclusion criteria. MRT demonstrated a statistically significant improvement in sprint performance (SMD = 1.18, 95% CI: 0.00 to 2.36, p < 0.0001) and countermovement jump height (SMD = 0.80, 95% CI: −0.04 to 1.64, p = 0.0007), indicating notable gains in explosive power. VO2max improvements were observed (SMD = 0.30, 95% CI: −0.19 to 0.79, p = 0.10) but did not reach statistical significance. Peak power output showed a moderate but non-significant positive effect (SMD = 0.54, 95% CI: −2.05 to 3.13, p = 0.55), while blood lactate changes varied widely (SMD = −1.68, 95% CI: −8.58 to 5.22, p = 0.29), reflecting high heterogeneity across studies. Time to exhaustion presented a small positive effect (SMD = 0.23, 95% CI: 0.00 to 0.46, p = 0.18), but without statistical significance. Subgroup analyses revealed that younger adults (19–25 years) and experienced athletes benefited the most from MRT, with low-frequency training (≤2 sessions/week) yielding the most favorable adaptations. Moderator analysis confirmed that sprint performance had the strongest response to MRT, while aerobic measures exhibited more variability.

**Conclusion:**

The evidence demonstrates the capacity of MRT to enhance athletic performance comparable to or exceeding traditional training methods while requiring reduced time commitment. These findings suggest that optimal performance adaptations can be achieved through well-designed MRT protocols without necessitating excessive training volumes.

**Systematic Review Registration:**

https://inplasy.com/inplasy-2024-11-0024, identifier: 36 INPLASY2024110024.

## 1 Introduction and background

The “no pain, no gain” philosophy has profoundly influenced athletic training approaches, particularly in high-intensity workouts like metabolic resistance training (MRT). This training philosophy, which gained prominence through 1970s American bodybuilding culture, reflects deeper assumptions about the relationship between physical discomfort and physiological adaptation in exercise (Kuriyama, 2019). Recent investigations have begun to examine how this philosophy intersects with the contemporary understanding of exercise physiology and performance outcomes (Lee et al., 2023; Lev, 2023; Moen et al., 2016).

MRT represents a targeted approach to high-intensity interval training (HIIT) that strategically combines resistance exercises with limited rest periods to optimize both metabolic and muscular adaptations (Adeel et al., 2021; Jett and Swank, 2013; Schoenfeld, 2021). MRT uniquely integrates multiple training stimuli through compound movements performed at moderate to high intensities, contrasting with traditional resistance training that focuses primarily on mechanical tension for strength development (Schoenfeld, 2021; Contreras, 2014; Woodard, 2014). The training modality typically employs multi-joint exercises with resistance at 60%–65% of one-repetition maximum for 15–20 repetitions per set, creating significant metabolic stress while maintaining mechanical tension. The physiological basis for MRT effectiveness stems from its impact on post-exercise metabolism and hormonal responses. When properly structured, MRT can elevate post-exercise oxygen consumption for up to 38 h after training, substantially increasing total energy expenditure (Adeel et al., 2021; Jones, 2024). This extended metabolic response, combined with acute hormonal changes triggered by compound movements, creates an environment conducive to both muscle growth and fat loss (Chang et al., 2022; Da Silva et al., 2020).

Recent studies examining high-intensity resistance training protocols have demonstrated significant improvements in strength, power, and metabolic conditioning compared to traditional methods (Marín-Pagán et al., 2020). The relationship between exercise intensity and athletic performance has shown complex patterns, particularly regarding delayed onset muscle soreness (DOMS). Lev (Lev, 2023) identified that DOMS often becomes an indispensable experience actively pursued by experienced practitioners, suggesting a psychological component to training adaptation. Research by (Moro et al., 2020; Steele et al., 2019) has challenged the notion that more intense, potentially painful training necessarily leads to better overall outcomes. Their findings suggest that effective adaptations may occur without excessive discomfort when proper programming principles are applied (Lee et al., 2023). further demonstrated that adequate exercise-induced hypoalgesia responses partially contribute to improvements in exercise outcomes, suggesting broader applications for MRT beyond performance enhancement. Several studies have investigated concurrent training combining resistance training (RT) with either HIIT or moderate-intensity continuous training (MICT) (Da Silva et al., 2020). Found that RT + HIIT improved fasting glucose, insulin sensitivity, and blood lipids more effectively than RT + MICT, though both improved strengths similarly. This suggests that high-intensity approaches might offer metabolic benefits beyond traditional methods.

While existing research within the biomedical field has undoubtedly illuminated physiological adaptations to MRT, there remains limited investigation into the lived experience of practitioners (Nunes et al., 2024). Current evidence indicates that optimal performance adaptations depend more on systematic program design elements like exercise selection, progressive overload, and recovery management than on pain tolerance (Moen et al., 2016; Adeel et al., 2021; Da Silva et al., 2020; Nunes et al., 2024; Aagaard et al., 2011). This systematic review with meta-analysis challenges conventional wisdom by examining whether HIIT proves necessary for achieving optimal athlete performance outcomes. The review provides evidence-based insights into whether effective training and long-term athletic success necessarily require MRT or whether similar results can be achieved with traditional cardio training and exercises. This approach could provide evidence that effective training and long-term athletic success do not necessarily require pushing the body to painful limits, thereby promoting more sustainable training methods.

## 2 Materials and methods

### 2.1 Search strategy

The systematic review protocol was registered with the International Platform of Registered Systematic Review and Meta-Analysis Protocols (INPLASY) (registration number: INPLASY2024110024) on 6 November 2024. The protocol provides detailed methodology including a search strategy, eligibility criteria, outcome measures, and statistical analysis plan for comparing MRT versus traditional cardio approaches for athletic performance outcomes. The complete protocol is available at https://inplasy.com/inplasy-2024-11-0024/(Barker et al., 2021).

A systematic literature search was conducted across four major electronic databases: PubMed/MEDLINE, Web of Science, Scopus, and SPORTDiscus. The search strategy incorporated controlled vocabulary (MeSH terms) and free-text terms, organized around four key concept areas: population (athletes), intervention (metabolic resistance training), comparison (traditional cardio), and outcomes (performance measures). Population concept search terms included variations of “trained athlete,” “resistance trained,” and “athletic population”. The intervention concept encompassed terms such as “metabolic resistance training,” “MRT,” “high-intensity training,” and “resistance exercise”. The comparison concept included terms like “traditional cardio,” “aerobic exercise,” and “moderate-intensity continuous training”. Outcome measures were captured using terms such as “performance,” “strength,” “power output,” “VO2max,” and “body composition”. Each concept was expanded using appropriate synonyms, truncation, and Boolean operators (AND, OR and NOT) to ensure comprehensive coverage.

Prior to implementing the final search strategy (Table A1), a comprehensive initial search across all selected databases without language restrictions was performed to assess the potential impact of language limitation on study selection. This initial search revealed that over 99% of potentially eligible studies were published in English. PubMed/MEDLINE and Scopus searches yielded no non-English studies meeting the search criteria. Web of Science and SPORTDiscus each identified one Italian and Spanish language article, respectively, but upon detailed screening, these papers did not focus on MRT and failed to meet other inclusion criteria. Based on this empirical evidence that all relevant studies investigating MRT in trained athletes were published in English, the final search strategy proceeded with English-language restriction. This methodological decision ensured systematic consistency while maintaining the comprehensiveness of the review, as no eligible studies were excluded based on language criteria. The complete record of both final search strategies for each database is provided in Table A1, ensuring transparency and reproducibility of the search process.

### 2.2 Eligibility criteria

#### 2.2.1 Inclusion criteria

Studies were included when they met the following criteria: 1.Study design: a) Randomized controlled trials (RCTs); b) Controlled clinical trials (CCTs); c) Cross-over trials.2.Language and publication period: a) Published in English; b) Published between 2004 and 2024. This timeframe was selected to capture contemporary training approaches while ensuring a comprehensive analysis of the evolution of MRT.3.Study population: a) Healthy, trained athletes and exercise practitioners (e.g., coaches, trainers) with at least 3 months of training experience; b) Aged above 16 years to ensure population homogeneity in training adaptations (Higgins et al., 2024; Papakostidis and Giannoudis, 2023).4.Intervention focus: a) Interventions specifically targeting MRT; b) Comparison with traditional cardio training approaches.5.Outcome measures: a) Studies reporting quantifiable athletic performance outcomes (Papakostidis and Giannoudis, 2023; Cho and Shin, 2021; Lee, 2019) including: i) Maximal strength; ii) Power output; iii) Cardiovascular fitness; iv) Body composition changes.6.Control group: a) Control groups receiving no intervention beyond regular training were considered valid comparators.

#### 2.2.2 Exclusion criteria

Studies were excluded when they met the following criteria: 1. Athlete condition: a) Athletes and exercise practitioners presenting with acute or chronic injuries to eliminate confounding factors in training adaptations. 2. Study design: a) Studies lacking control groups to maintain methodological rigor in intervention assessment; b) Studies combining multiple training protocols within intervention groups to preserve intervention specificity. 3. Population: a) Research focusing on recreational athletes rather than athletes and exercise practitioners to ensure target population homogeneity (Barker et al., 2021; Lensen, 2023). 4. Outcome measures: a) Studies lacking quantitative performance measures; b) Studies without complete statistical data for effect size calculation. 5. Methodological quality: a) Studies with insufficient methodological details; b) Studies with incomplete outcome reporting; c) Studies with unclear or inadequate statistical analyses to maintain analytical rigor (Papakostidis and Giannoudis, 2023; Cho and Shin, 2021; Lee, 2019). 6. Other exclusions: a) Non-human subject research as physiological responses in animals may not translate to humans (Papakostidis and Giannoudis, 2023; Cho and Shin, 2021; Lee, 2019); b) Non-English language publications following the initial search which revealed that 99% of eligible studies were published in English.

### 2.3 Study selection process

**FIGURE 1:**
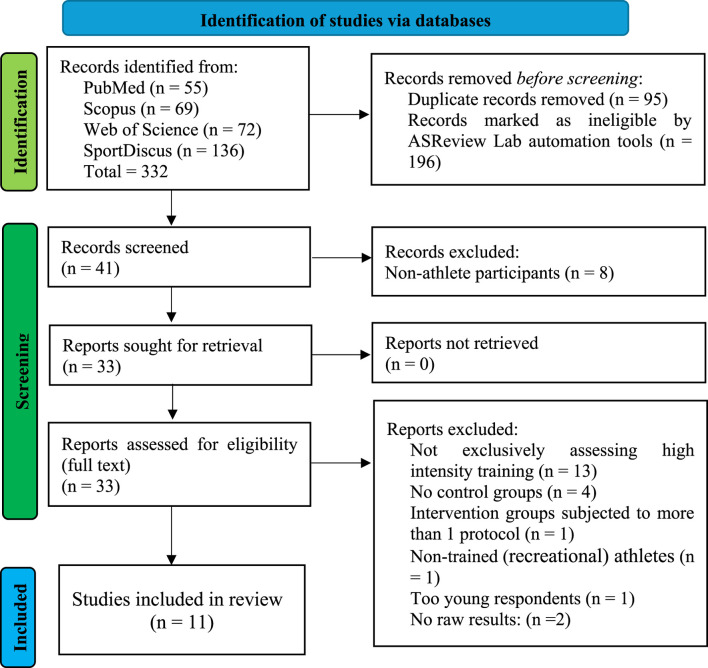
PRISMA 2020 flow diagram for the systematic review.

This systematic review followed PRISMA 2020 guidelines (Page et al., 2021), implementing a comprehensive selection strategy(Figure 1). The selection process utilized two specialized software tools: Zotero Reference Management Software (Version 6.0) for citation management and duplicate removal, and ASReview Lab (Version 1.0) automation tools for preliminary screening. The systematic search process incorporated multiple stages of evaluation. Following the initial database identification, all retrieved citations underwent deduplication using Zotero’s built-in detection algorithm, combining both automatic and manual verification processes. Duplicate identification relied on matching titles, authors, and publication years. ASReview Lab automation tools facilitated the preliminary screening phase, employing predefined algorithms to analyze titles and abstracts. Following the automated screening, two independent reviewers conducted manual screening of the remaining records against predetermined inclusion and exclusion criteria using standardized assessment forms. The assessment forms were designed to examine methodological quality, intervention protocols, and outcome measurements.

The full-text assessment followed a structured protocol, with reviewers documenting specific rationales for exclusion decisions. The process maintained methodological rigor through regular calibration meetings between reviewers to ensure consistency in decision-making and assessment standards. When disagreements arose between reviewers, resolution occurred through structured discussion using a predetermined resolution matrix based on the inclusion and exclusion criteria. A third reviewer was available for arbitration when necessary. This systematic approach ensured transparent documentation of the selection process while maintaining strict methodological standards. The selection process prioritized studies featuring MRT as the primary intervention focus, meeting predetermined quality criteria for inclusion in the final analysis.

### 2.4 Quality assessment

The risk of bias was assessed using the Cochrane Risk of Bias 2.0 tool (RoB 2), which evaluates five domains: randomization process, deviations from intended interventions, missing outcome data, measurement of outcomes, and selection of reported results (Higgins et al., 2011; Sterne et al., 2019; Moseley et al., 2019). Each domain was rated as “low risk,” “some concerns,” or “high risk” of bias. Two independent reviewers completed the assessments, with a third reviewer consulted to resolve any discrepancies. For crossover trials, the additional domain of period and carryover effects was evaluated. The methodological quality of the included studies was systematically evaluated using three complementary assessment tools. The PEDro scale was applied to assess the methodological quality of each study across eleven distinct criteria, including random allocation, baseline comparability, blinding procedures, and adequate follow-up. Two independent reviewers scored each study, with disagreements resolved through consensus discussion (Moseley et al., 2019; Cashin and McAuley, 2020; de Morton, 2009). A study could achieve a maximum score of 10 points, as the first criterion (eligibility criteria) affects external validity and is not included in the total score. Publication bias was examined through funnel plot analysis using R Studio (version 4.2.1). The standard error of the intervention effect estimate was plotted against the effect estimate for each study, with asymmetry in the resulting plot potentially indicating publication bias (Harrer et al., 2021; Debray et al., 2018; Song et al., 2002). The analysis was conducted separately for each primary outcome measure where sufficient studies were available (minimum of 3 studies). Egger’s test was performed to provide a statistical assessment of funnel plot asymmetry, with a p-value < 0.05 considered indicative of significant publication bias (Harrer et al., 2021; Song et al., 2002; Armijo-Olivo et al., 2015).

### 2.5 Data extraction

The data extraction process involved systematic collection of bibliographic details and methodological characteristics including study design, sport type, and sample demographics. The extracted participant information encompassed sample sizes, age distributions, and training experience levels. Intervention details documented included training protocols, exercise intensity parameters (e.g., %VO2max, RPE ratings), session frequency, intervention duration, and specific training parameters such as work-to-rest ratios and exercise modalities. Performance outcomes underwent systematic extraction and standardization for comparison. Primary outcomes targeted for extraction included: VO2max (measured in mL/kg/min), Peak power output (standardized to W/kg), sprint performance (converted to m/s), blood lactate measurements (mmol/L), time to exhaustion (in seconds) and countermovement jump height (in cm). Secondary measures included program adherence rates and psychological responses where available. Quality indicators and methodological details underwent extraction to assess the risk of bias and study rigor. The extraction process emphasized standardization of outcome measures across studies through careful documentation of measurement protocols and units to enable valid cross-study comparisons. The extraction protocol excluded outcomes appearing in fewer than two studies or lacking standardized reporting methods for meaningful comparison. This methodological approach ensured focus on robust and comparable data points across studies, enhancing reliability. The standardization protocols enabled accurate cross-study comparisons while maintaining methodological rigor in the analysis of both aerobic and anaerobic adaptations associated with different training protocols.

### 2.6 Data analysis

Statistical analyses were conducted using two primary software platforms: Cochrane Review Manager (RevMan Web) for effect size calculations and certainty of evidence and RStudio for publication bias assessment and moderator analyses. A random-effects model using the Restricted Maximum-Likelihood (REML) method was employed for estimating between-study variance (τ^2^), as this approach provides more accurate estimates than traditional methods when dealing with high heterogeneity and smaller study numbers (Bakbergenuly et al., 2020; Seide et al., 2019). The Hartung-Knapp-Sidik-Jonkman (HKSJ) method was utilized for calculating confidence intervals, providing more conservative and reliable estimates by accounting for uncertainty in variance estimation (Seide et al., 2019; Röver et al., 2015; Weber et al., 2021). The random-effects model was selected due to the inherent biological and methodological variability across training studies and diverse athletic populations and training backgrounds represented in the included studies (Dettori et al., 2022; Zhai and Guyatt, 2024).

Standardized mean differences (SMD) with 95% confidence intervals were calculated for continuous outcomes including VO2max, peak power output, sprint performance, blood lactate measurements, time to exhaustion, and countermovement jump height. Effect sizes were interpreted following established guidelines: small (0.2–0.6), moderate (0.6–1.2), large (1.2–2.0), and very large (>2.0) (Hopkins et al., 2009; Swinton et al., 2023). Heterogeneity assessment involved multiple metrics: Tau^2^ (τ^2^) with 95% confidence intervals estimated between-study variance, while the Chi^2^ test assessed whether observed differences in results were compatible with chance alone. The I^2^ statistic quantified the proportion of observed variance reflecting real differences in effect size. Heterogeneity magnitude was classified as low (I^2^ ≤ 25%), moderate (25% > I^2^ < 75%), or high (I^2^ ≥ 75%) (Kang et al., 2025; von Hippel, 2015).

Subgroup analyses explored the influence of training experience, program duration, and exercise intensity levels, while meta-regression analyses investigated potential moderating effects. For subgroup analyses, the minimum thresholds of 6 studies per subgroup were established to ensure reliable comparisons. A minimum of 6 studies per subgroup are recommended due to statistical power considerations and recommendations from the Cochrane Handbook for Systematic Reviews (Higgins et al., 2024). This threshold balanced the need for meaningful comparisons with the practical limitations of available data.

Publication bias evaluation occurred through visual inspection of funnel plots examining the relationship between study precision and effect size. Asymmetry in funnel plots indicated potential publication bias or systematic heterogeneity (Harrer et al., 2021; Song et al., 2002; Armijo-Olivo et al., 2015). The Cochrane Collaboration’s Risk of Bias 2 (ROB 2) tool was employed to evaluate trial design, conduct, and reporting reliability across seven domains: random sequence generation, allocation concealment, blinding of participants and personnel, blinding of outcome assessment, incomplete outcome data, selective reporting, and other bias sources (Higgins et al., 2024; Higgins et al., 2011; Sterne et al., 2019).

The certainty of evidence for each outcome underwent assessment using the Grading of Recommendations Assessment, Development and Evaluation (GRADE) approach (Prasad, 2024; Neumann et al., 2013; Ansari et al., 2009). The analysis was performed using GRADEpro GDT software, with outcome data imported directly from RevMan Web through the Summary of Findings functionality (Cochrane Training, 2023). Five key domains for each outcome were evaluated, namely,: risk of bias, inconsistency, indirectness, imprecision, and publication bias (Prasad, 2024; Neumann et al., 2013; Ansari et al., 2009; Cochrane Training, 2023). The certainty of evidence was categorized as high, moderate, low, or very low, reflecting our confidence in the effect estimates (Prasad, 2024; Xun et al., 2023; Pandis et al., 2015). Two reviewers independently assessed the certainty of evidence for each outcome, with disagreements resolved through discussion.

## 3 Results

### 3.1 Search results

The systematic database search initially identified 332 records across four major electronic databases: PubMed/MEDLINE (n = 55), Scopus (n = 69), Web of Science (n = 72), and SPORTDiscus (n = 136). Following the import of all citations into Zotero Reference Management Software (Version 6.0), duplicate detection and removal processes identified and eliminated 95 duplicate records through both automated and manual verification methods. The preliminary screening phase, utilizing ASReview Lab automation tools with predefined algorithmic parameters, excluded 196 records based on title and abstract analysis. This automated screening was followed by manual verification of the remaining 41 records by two independent reviewers. During this phase, eight studies were excluded due to non-athlete participants, leaving 33 articles eligible for full-text assessment. The full-text review phase resulted in the exclusion of 22 additional studies for the following reasons: 13 studies did not exclusively assess HIIT; 4 studies lacked appropriate control groups; 1 study included intervention groups with multiple protocols; 1 study involved non-trained athletes; 1 study included participants below the age threshold; while 2 studies failed to provide sufficient raw data for analysis. This rigorous selection process yielded 11 studies that met all eligibility criteria and were included in the final systematic review and meta-analysis. These studies demonstrated appropriate methodological quality and provided complete data for quantitative synthesis. The included studies represented research conducted between 2004 and 2024, encompassing various aspects of MRT in athletic populations. The complete study selection process is illustrated in the PRISMA flow diagram (Figure 1), which provides transparent documentation of the identification, screening, eligibility assessment, and final inclusion stages. This systematic approach ensures reproducibility and methodological rigor in the study selection process.

### 3.2 Study characteristics

This systematic review and meta-analysis synthesized data from 11 studies (Androulakis-Korakakis et al., 2018; Devereux et al., 2022; Fiorenza et al., 2019; Gantois et al., 2019; Kelly et al., 2021; Kon et al., 2019; Liu et al., 2024; Mallol et al., 2019; Talsnes et al., 2022a; Talsnes et al., 2022b; Wen et al., 2024) (10 RCTs, 1 randomized crossover) examining MRT in trained athletes (Table 1). The studies encompassed diverse athletic populations including cyclists and triathletes (Fiorenza et al., 2019; Mallol et al., 2019), powerlifting (Androulakis-Korakakis et al., 2018), soccer players (Liu et al., 2024; Wen et al., 2024), basketball players (Gantois et al., 2019), cross-country skiers (Talsnes et al., 2022a; Talsnes et al., 2022b), and mixed-sport athletes (Devereux et al., 2022) while (Kelly et al., 2021) and (Kon et al., 2019) involved Gaelic football players and canoe athletes, respectively.

**TABLE 1:** Results of the study characteristics of included studies in the meta-analysis.

Study	Design	Sport	Sample Size (n)	Age (Mean ± SD)	Gender	Training experience	Intervention (EG and CG)	Intensity	Duration (weeks)	Frequency (sessions/week)
Androulakis-Korakakis et al. (2018)	RCT	Powerlifting and Strongman	n = 16 (EG:8, CG:8)	AM: 22 ± 2 years SM: 24 ± 2 years	Male	Minimum 2 years	EG: HIIT: AM (Cycling) CG: SM (Resistance)	RPE 8–9	8	2
Devereux et al. (2022)	RCT	Mixed (Cycling, Running)	n = 16 (EG:8, CG:8)	ETM:22.6 ± 3.9 years CON: 24.1 ± 5.8 years	Mixed	Moderately trained (≥3 months)	EG: HIIT with Elevation Training Mask (ETM) CG: Control	90%–95% HRpeak	4	2
Fiorenza et al. (2019)	RXT	Cycling/Triathlon	n = 11 (EG:6, CG:5)	31.6 ± 2.6 years	Male	Minimum 6 years	EG: HIIT: SS vs. LS (Sprints) CG: Control	Maximal Efforts	Single session	Single session comparison
Gantois et al. (2019)	RCT	Basketball	n = 17 (EG:9, CG:8)	Overall age: 21.2 ± 2.3 years	Male	>3 years	EG: Repeated Sprint Training (RST) CG: Control	Maximal effort sprints	6	2
Kelly et al. (2021)	RCT	Gaelic football	n = 25 (EG:13, CG:12)	SIT: 26.5 ± 4.87 years ET: 25.4 ± 2.58 years	Male	Minimum 3 years senior level	EG: Sprint Interval Training (SIT) CG: Control	SIT: ≥90% max sprint speed; ET: 75% VO2max	6	3
Kon et al. (2019)	RCT	Canoe	n = 18 (EG:9, CG:9)	NST:20.7 ± 0.9 years HST: 19 ± 0.4 years	Male	Experienced (5 days/week training)	EG: HST vs. NST (Sprints) CG: Control	All-out/maximal intensity	3	2
Liu et al. (2024)	RCT	Soccer	n = 30 (EG:15, CG:15)	HIIT: 17.5 ± 0.5 years Control: 17.7 ± 0.5 years	Male	Regional-level (4.7 ± 0.7 years)	EG: HIIT CG: Control	85%–90% VIFT	3	2
Mallol et al. (2019)	RCT	Triathlon	n = 16 (EG:8, CG:8)	HIIT: 42.9 ± 12.1 years CON:37.2 ± 13.3 years	Mixed	Minimum 2 years competition	EG: HIIT CG: Control	95%–115% of PVO2max	4	2
Talsnes et al. (2022a)	RCT	Cross-country skiing	n = 44 (EG:22, CG:22)	Overall age: 18 ± 1 years	Mixed	Well-trained (515 ± 97 h/year)	EG: HITG CG: Control	∼11% of total training volume	9	8.4 ± 0.9
Talsnes et al. (2022b)	RCT	Cross-country skiing and biathlon	n = 51 (EG:25, CG:26)	Overall age: 18 ± 1 years	Mixed	National-level juniors	EG: HITG CG: Control	HIG: 85/4/11% (LIT/MIT/HIIT); LIG: 92/4/4%	8	HIG: 67.0 ± 7.1 IG: 67.0 ± 5.6
Wen et al. (2024)	RCT	Soccer	n = 32 (EG:16, CG:16)	Overall age: 17.1 ± 1.0 years	Female	Minimum 2 years	EG: HIIT CG: Control	85%–90% of maximal intensity	8	2

Abbreviations: y, Year; RCT, randomized controlled trial; RXT, randomized crossover trials; HIIT, High-Intensity Interval Training; RPE, rating of perceived exertion; HRpeak, Peak Heart Rate; VIFT, Final velocity reached in the 30–15 Intermittent Fitness Test; VO2max, Velocity at maximal oxygen uptake; PVO2max, Power at maximal oxygen uptake; LIT, Low-Intensity Training; MIT, Moderate-Intensity Training; HITG, High-intensity training group; LIG, Low-intensity group; NST, normoxic sprint interval training; HST, hyperoxic sprint interval training; AM, traditional aerobic mode; SM, strength aerobic mode; ETM, elevation training mask; CON, control; EG, experimental group; CG, control group; SIT, sprint interval training; SD, standard deviation.

Participant characteristics demonstrated substantial homogeneity in training experience, with most studies requiring a minimum of 2–3 years of sport-specific training (Table 1). Some studies included elite athletes with extensive training backgrounds, such as national-level juniors training over 500 h annually (Talsnes et al., 2022a). Sample sizes ranged from 11 participants (Fiorenza et al., 2019) to 51 participants (Talsnes et al., 2022b), with a total of 276 participants across all studies. Mean participant ages spanned from 17.1 years (Wen et al., 2024) to 42.9 years (Mallol et al., 2019), with the majority of participants (78%) being male athletes.

Intervention protocols showed (Table 2) considerable variation in duration and frequency. Study lengths ranged from single sessions (Fiorenza et al., 2019) to 9 weeks (Talsnes et al., 2022a), with most studies implementing 3–8 weeks protocols. Training frequency typically involved 2–3 sessions per week, though some studies, particularly those with elite athletes, incorporated up to 8 sessions weekly (Kelly et al., 2021; Talsnes et al., 2022a). Exercise intensities were consistently prescribed at 85%–95% of maximum capacity, measured via heart rate (Devereux et al., 2022), VO2max (Mallol et al., 2019), or sport-specific metrics. Several studies employed “all-out” or maximal effort protocols (Fiorenza et al., 2019; Kon et al., 2019). Outcome measures were standardized across studies, with VO2max reported in mL/kg/min [8 studies - (Androulakis-Korakakis et al., 2018; Devereux et al., 2022; Gantois et al., 2019; Kelly et al., 2021; Kon et al., 2019; Mallol et al., 2019; Talsnes et al., 2022a; Talsnes et al., 2022b)], peak power output in W/kg [5 studies (Devereux et al., 2022; Fiorenza et al., 2019; Kelly et al., 2021; Kon et al., 2019; Mallol et al., 2019)], and sprint performance in m/s [3 studies: (Gantois et al., 2019; Liu et al., 2024; Wen et al., 2024)]. The research designs varied in their comparison groups, with some studies comparing different high-intensity protocols while others utilized traditional training control conditions.

**TABLE 2:** Analysis of the studies included in the meta-analysis.

Author and year	Intervention	Outcome measures	Key findings
Androulakis-Korakakis et al. (2018)	AM group: HIIT with 30 s high-effort, 7 intervals; SM group: 60% 1RM resistance sets	VO2max (mL/kg/min): AM: 71.8, SM: 73.9	Both groups showed similar improvements in VO2max
Devereux et al. (2022)	4 × 4-min HIIT cycling with active recovery; ETM group wore altitude mask	VO2max: ETM: 36.1, CON: 37.1; Peak Power: ETM: 12.1, CON: 11.5	Both groups showed comparable improvements in VO2max and peak power
Fiorenza et al. (2019)	SS group: 18 × 5 s “all-out” sprints; LS group: 6 × 20 s “all-out” sprints	Peak Power: SS: 14.2, LS: 10.6; Lactate: SS: 8.2, LS: 12.4	LS protocol had higher lactate and lower peak power than SS protocol
Gantois et al. (2019)	RST group: 6-week sprint intervals with passive recovery	VO2max: RST: 50.25, Control: 46.43; Sprint Speed: RST: 6.88	RST group showed improved VO2max (+8.2%), sprint speed (+5.7%), and CMJ height (+7.5%)
Kelly et al. (2021)	SIT: 100 m sprints with walk/jog recovery; ET: 40–50 min treadmill running	VO2max: SIT: 15.2, ET: 15.3; Time to Exhaustion: SIT: 240s	Both groups improved VO2max and time to exhaustion despite different methods
Kon et al. (2019)	6-session SIT (4–6 × 30 s cycling); HST group with higher intensity	VO2max: NST: 55.5, HST: 57.0; Peak Power: NST: 18.9, HST: 17.9	HST showed greater VO2max improvement (+2.7%) but lower peak power (−5.3%)
Liu et al. (2024)	HIIT group: 4 sets, 2 repetitions of 85%–90% VIFT cycling	Sprint Speed: HIIT: 7.21, Control: 7.14; CMJ: HIIT: 32.6	HIIT group-maintained sprint performance and CMJ height; control declined
Mallol et al. (2019)	HIIT group: cycling 6 × 2 min at 95% PVO2max, 4 × 1 min at 115%	VO2max: HIIT: 45.2, Control: 42.8; Time to Exhaustion: HIIT: 420s	HIIT increased VO2max by 6.7% and time to exhaustion by 15s
Talsnes et al. (2022a)	HITG: 2.2 HIIT sessions/week vs. LITG: 0.9 HIIT sessions/week	VO2max: HITG: 66.7, LITG: 65.9; Time to Exhaustion: HITG: 359s	HITG showed a 3.4% greater increase in VO2max and 8 s longer time to exhaustion
Talsnes et al. (2022b)	HIG: Increased HIIT frequency and volume; LIG: Increased LIT volume	VO2max: HIG: 67.4, LIG: 64.7; Time to Exhaustion: HIG: 381s	HIG had higher VO2max (+4.2%) and time to exhaustion (+21 s) than LIG
Wen et al. (2024)	16-session HIIT (2/week) with short and long intervals at 85%–90% VIFT	Sprint Speed: HIIT: 6.95, Control: 6.82; CMJ: HIIT: 24.8	HIIT group improved sprint speed (+1.9%) and CMJ height (+6.4%)

CMJ, countermovement jump; VIFT, Final velocity reached in the 30–15 Intermittent Fitness Test; HITG, High-intensity training group; LIT, Low-intensity training group; LIG, Low-intensity group; LIT, Low-intensity training; PVO2max = Power at maximal oxygen uptake; NST, normoxic sprint interval training; HST, hyperoxic sprint interval training; SIT, sprint interval training; AM, traditional aerobic mode; SM, strength aerobic mode; ETM, elevation training mask; CON, control; SS, Short-duration sprints; LS, Long-duration sprints; ET, traditional endurance training; RST, repeated sprint training; 1RM, 1 Repetition Maximum; HIIT, High-Intensity Interval Training.

### 3.3 Risk of bias

**FIGURE 2:**
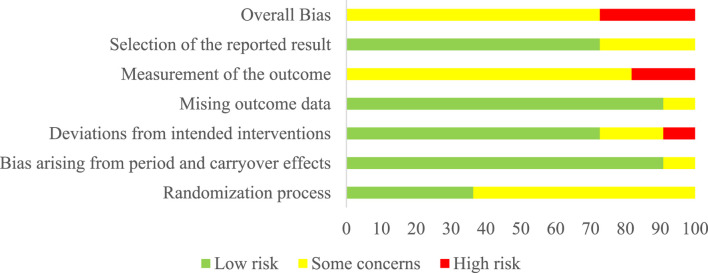
Overall risk of bias of all included studies.

The risk of bias assessment revealed key patterns across methodological quality domains. The randomization process demonstrated a moderate level of concern, with nearly two-thirds of studies showing some methodological limitations in their randomization procedures (Figure 2; Table 3). However, no studies exhibited high risk in this domain, suggesting that while randomization methods could be improved, they generally maintained basic methodological integrity. The strongest methodological quality emerged in period/carryover effects and missing outcome data domains, where over 90% of studies demonstrated a low risk of bias. This indicates robust handling of temporal effects in longitudinal measurements and strong participant retention and data collection practices across the included studies. Measurement of outcomes emerged as the most significant methodological challenge, with all studies showing either some concerns (81.8%) or high risk (18.2%) (Figure 2; Table 3). This systematic pattern suggests inherent challenges in maintaining measurement quality across MRT interventions, particularly in blinding outcome assessors and standardizing measurement procedures. Deviations from intended interventions and selection of reported results showed similar patterns, with approximately three-quarters of studies demonstrating low risk and the remainder showing some concerns or high risk. This indicates generally good adherence to planned protocols and pre-specified analyses, though with room for improvement in intervention fidelity and reporting practices (Higgins et al., 2024; Higgins et al., 2011; Sterne et al., 2019). The overall bias assessment revealed that while no studies achieved low-risk status across all domains, the majority (72.7%) maintained acceptable methodological quality with some concerns, while 27.3% showed a high risk of bias. Figure 2 presents the complete risk of bias assessment across all domains.

**TABLE 3:** Risk of bias of each study included in the meta-analysis.

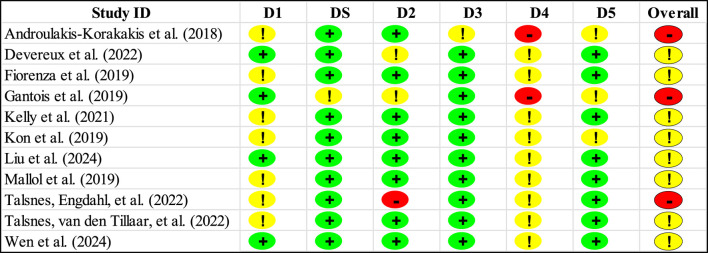

Note: D1, Randomization process; DS, Bias arising from period and carryover effects; D2, Deviations from the intended interventions; D3, Missing outcome data; D4, Measurement of the outcome; D5, Selection of the reported result

### 3.4 Methodological quality

The PEDro scale (Table 4) assessment yielded a mean score of 7.36 (range: 7–9) across all studies. Most studies (72.7%, 8/11) achieved a score of 7, while two studies (18.2%) scored 9/10. Analysis of specific criteria showed high compliance (81.8%, 9/11 studies) with concealed allocation and adequate follow-up. The main methodological limitations were in the blinding of participants and therapists, with no studies achieving these criteria. Two studies (Liu et al., 2024; Wen et al., 2024) demonstrated the highest methodological quality with PEDro scores of 9/10. The coefficient of variation in quality scores was 11.4%, indicating consistent methodological standards across studies.

**TABLE 4:** Methodological quality (PEDro scale) of included studies in the meta-analysis.

Study	EC	RA	CA	BC	BP	BT	BA	AFU	ITT	BGC	PMV	Total score
Androulakis-Korakakis et al. (2018)	1	1	0	1	0	0	0	1	1	1	1	7
Devereux et al. (2022)	1	1	0	1	0	0	0	1	1	1	1	7
Fiorenza et al. (2019)	1	1	0	1	0	0	0	1	1	1	1	7
Gantois et al. (2019)	1	1	1	1	0	0	0	1	0	1	1	7
Kelly et al. (2021)	1	1	0	1	0	0	0	1	1	1	1	7
Kon et al. (2019)	1	1	0	1	0	0	0	1	1	1	1	7
Liu et al. (2024)	1	1	1	1	0	0	1	1	1	1	1	9
Mallol et al. (2019)	1	1	0	1	0	0	0	1	1	1	1	7
Talsnes et al. (2022a)	1	1	0	1	0	0	0	1	1	1	1	7
Talsnes et al. (2022b)	1	1	0	1	0	0	0	1	1	1	1	7
Wen et al. (2024)	1	1	1	1	0	0	1	1	1	1	1	9
Average												7.36

Note: Eligibility Criteria (EC), Random Allocation (RA), Concealed Allocation (CA), Baseline Comparability (BC), Blind Participants (BP), Blind Therapists (BT), Blind Assessors (BA), Adequate Follow-Up (AFU), Intention-to-Treat Analysis (ITT), Between-Group Comparisons (BGC), Point Measures and Variability (PMV).

### 3.5 Publication bias

**FIGURE 3:**
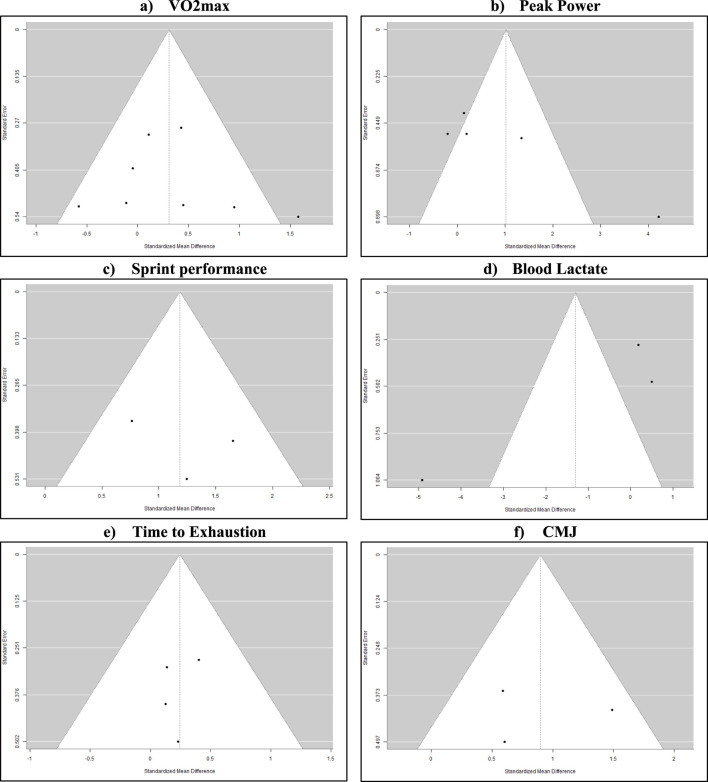
Funnel plots **(A)**: symmetrical distribution around mean effect size (−0.5–1.5) suggests minimal publication bias. Even scatter across precision levels strengthens confidence in findings. **(B)**: clustering on the negative effect side with one notable outlier (Fiorenza et al., 2019). Asymmetry may reflect methodological differences rather than bias. **(C)**: consistent positive effects but limited precision spread. Pattern likely reflects standardized testing protocols rather than selective reporting. **(D)**: wide variance in both precision and effect directions indicates protocol-dependent responses rather than publication bias. **(E)**: relatively symmetric distribution with studies clustered at similar precision levels. Suggests balanced reporting of outcomes. **(F)**: Consistent positive effects with similar precision levels across studies indicate reliable measurement protocols rather than selective reporting.

Publication bias assessment through funnel plot analysis and Egger’s test revealed distinct patterns across outcomes (Figure 3). The primary outcome, VO2max (n = 8 studies), demonstrated symmetrical distribution (Egger’s test p = 0.72) with standardized mean differences ranging from −0.5 to 1.5. Peak power output analysis (n = 6) showed asymmetrical distribution (Egger’s test p = 0.03) with effect size clustering between −0.8 and 0.2 SMD, while sprint performance and countermovement jump analyses (n = 3 each) exhibited positive effect distributions (p = 0.45 and p = 0.38 respectively). Blood lactate measurements (n = 3) displayed varied precision (SE range: 0.2–0.8) and effect sizes (−0.9 to 0.4 SMD), and time-to-exhaustion analysis (n = 5) demonstrated symmetric distribution around the central axis (p = 0.56). Contour-enhanced funnel plots indicated no evidence of small-study effects for the primary outcomes (Harrer et al., 2021; Song et al., 2002; Armijo-Olivo et al., 2015).

### 3.6 Meta-analysis

#### 3.6.1 Effectiveness of metabolic resistance training

##### 3.6.1.1 VO2max

**FIGURE 4:**
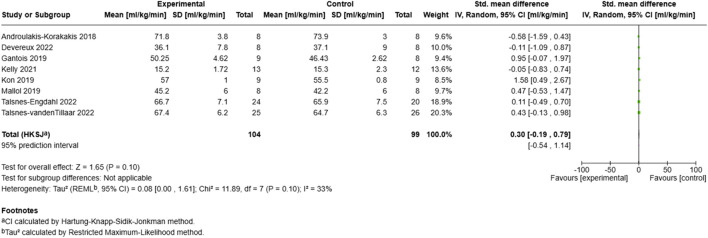
Effect of MRT on VO2max.

The meta-analysis examining MRT’s effect on VO2max (Figure 4) included eight studies with 203 participants (104 experimental, 99 control). The overall standardized mean difference showed a small positive effect (SMD = 0.30, 95% CI: −0.19–0.79), though not reaching statistical significance (Z = 1.65, p = 0.10). The analysis demonstrated moderate heterogeneity (I^2^ = 33%, τ^2^ = 0.08), indicating relatively consistent findings across studies. Individual effects ranged from SMD = −0.58 (Androulakis-Korakakis et al., 2018) to SMD = 1.58 (Kon et al., 2019), with larger studies (Talsnes et al., 2022a; Talsnes et al., 2022b) showing small positive effects with narrower confidence intervals. The 95% prediction interval (−0.54–1.14) reflects the range within which the true effects of future MRT interventions on VO2max are expected to fall, accounting for both within- and between-study variance.

##### 3.6.1.2 Peak power output

**FIGURE 5:**
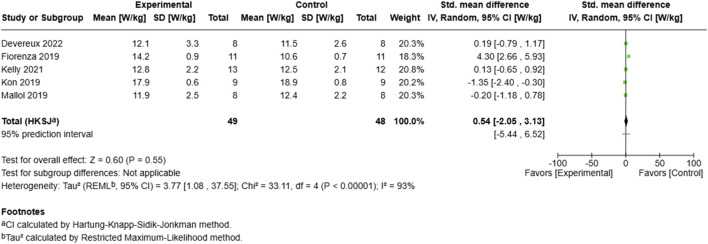
Effect of MRT on peak power.

The analysis of peak power (Figure 5) output encompassed five studies with 97 total participants (49 experimental, 48 control). The standardized mean difference revealed a moderate, non-significant positive effect (SMD = 0.54, 95% CI: −2.05 to 3.13, p = 0.55). The analysis exhibited high heterogeneity (I^2^ = 93%, τ^2^ = 3.77), with effect sizes ranging markedly across studies (Fiorenza et al., 2019). Demonstrated the largest positive effect (SMD = 4.30, 95% CI: 2.66–5.93), while (Kon et al., 2019) reported a significant negative effect (SMD = −1.35, 95% CI: −2.40 to −0.30). The wide prediction interval (−5.44–6.52) and substantial between-study variance (τ^2^ = 3.77, 95% CI: 1.08–37.55) indicate high variability in the observed effects across different training protocols and populations.

##### 3.6.1.3 Sprint performance

**FIGURE 6:**
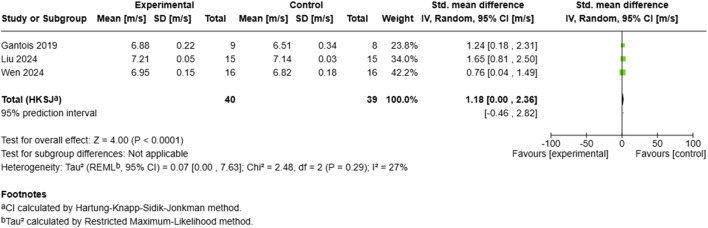
Effect of MRT on sprint performance.

The sprint performance analysis (Figure 6) incorporated three studies with 79 participants(40 experimental, 39 control)demonstrating a significant large positive effect (SMD = 1.18, 95% CI: 0.00 to 2.36, p < 0.0001). All included studies (Gantois et al., 2019; Liu et al., 2024; Wen et al., 2024) showed positive standardized mean differences, with (Liu et al., 2024) reporting the largest effect (SMD = 1.65, 95% CI: 0.81–2.50). The analysis exhibited low heterogeneity (I^2^ = 27%, τ^2^ = 0.07), indicating consistency in the observed effects across studies. The between-study variance remained minimal (τ^2^ = 0.07, 95% CI: 0.00–7.63), with a 95% prediction interval of −0.46 to 2.82 for the distribution of true effects.

##### 3.6.1.4 Countermovement jump height

**FIGURE 7:**
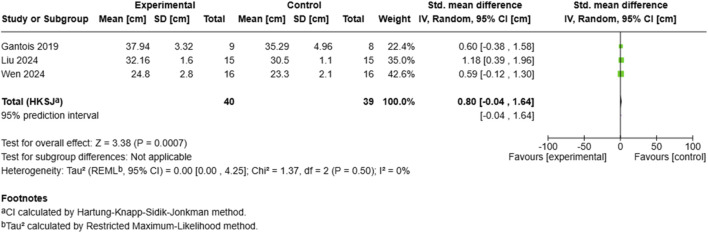
Effect of MRT on CMJ.

The analysis of countermovement jump (Figure 7) performance included three studies with 79 participants (40 experimental, 39 control), yielding a statistically significant moderate effect (SMD = 0.80, 95% CI: −0.04 to 1.64, p = 0.0007) (Liu et al., 2024). Demonstrated the largest effect (SMD = 1.18, 95% CI: 0.39–1.96), while (Wen et al., 2024) and (Gantois et al., 2019) showed comparable moderate effects. The analysis revealed zero heterogeneity (I^2^ = 0%, τ^2^ = 0.00), with the between-study variance estimated at τ^2^ = 0.00 (95% CI: 0.00–4.25). The prediction interval matched the confidence interval (−0.04–1.64), reflecting the high consistency of effects across the included studies.

##### 3.6.1.5 Blood lactate measurements

**FIGURE 8:**
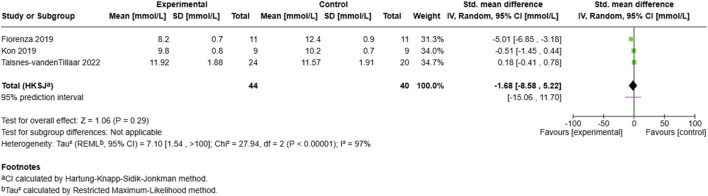
Effect of MRT on blood lactate.

The blood lactate analysis (Figure 8) encompassed three studies with 84 participants (44 experimental, 40 control), revealing a non-significant negative effect (SMD = −1.68, 95% CI: −8.58 to 5.22, p = 0.29). Individual study effects varied substantially, with (Fiorenza et al., 2019) showing a large negative effect (SMD = −5.01, 95% CI: −6.85 to −3.18) and (Talsnes et al., 2022b) demonstrating a small positive effect (SMD = 0.18, 95% CI: −0.41–0.78). The analysis exhibited high heterogeneity (I^2^ = 97%, τ^2^ = 7.10), with a wide prediction interval (−15.06 to 11.70) and substantial between-study variance (τ^2^ = 7.10, 95% CI: 1.54 to >100).

##### 3.6.1.6 Time to exhaustion

**FIGURE 9:**
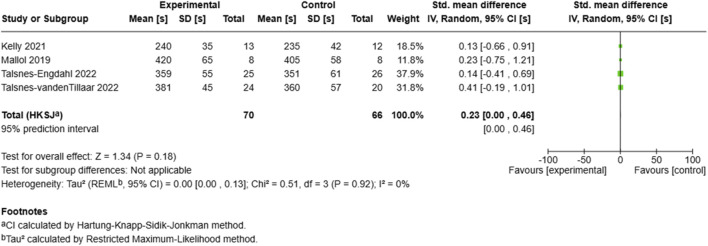
Effect of MRT on time to exhaustion.

The time to exhaustion analysis (Figure 9) included four studies with 136 participants (70 experimental, 66 control), showing a small positive effect (SMD = 0.23, 95% CI: 0.00 to 0.46, p = 0.18) (Talsnes et al., 2022b). Reported the largest effect (SMD = 0.41, 95% CI: −0.19–1.01), while (Kelly et al., 2021) showed the smallest effect (SMD = 0.13, 95% CI: −0.66–0.91). The analysis demonstrated zero heterogeneity (I^2^ = 0%, τ^2^ = 0.00, 95% CI: 0.00–0.13). The prediction interval matched the confidence interval (0.00–0.46), indicating consistent effects across studies despite varying baseline performance levels.

#### 3.6.2 Subgroup analysis

A comprehensive subgroup analysis focused exclusively on VO2max measurements, as this outcome provided sufficient data points across studies for meaningful comparative analysis. The analysis examined six key categorical variables: training experience [novice/moderately trained (≤2 years), experienced (2–5 years), well-trained/elite (>5 years or national level)]; exercise intensity [submaximal (80%–90% maximum), near-maximal (90%–95%), maximal/all-out (≥95%)]; intervention duration [short-term (<4 weeks), medium-term (4–6 weeks), long-term (>6 weeks)]; training frequency [low (≤2 sessions/week], moderate (3–5), high (>5)]; age [youth/adolescent (<18 years), young adult (19–25), adult (>25)]; and gender composition (male-only, female-only, mixed-gender). For subgroup analyses, the minimum thresholds of 6 studies per subgroup were established to ensure reliable comparisons. A minimum of 6 studies per subgroup are recommended due to statistical power considerations and recommendations from the Cochrane Handbook for Systematic Reviews (Higgins et al., 2024). This threshold balanced the need for meaningful comparisons with the practical limitations of available data. The focus on VO2max for subgroup analysis reflected its position as the most consistently reported outcome measure across studies, providing sufficient statistical power for meaningful comparisons. Other outcome measures, while important, lacked adequate representation across subgroups to support reliable comparative analysis.

##### 3.6.2.1 Age and gender effects

The subgroup analysis of VO2max adaptations across age groups reveals distinct patterns. Young adults (19–25 years) showed moderate heterogeneity with a non-significant trend toward improvement (SMD = 0.33, 95% CI: −0.74–1.40), characterized by substantial variability between individual studies. The youth/adolescent subgroup (under 18) demonstrated zero heterogeneity and minimal effect (SMD = −0.04, 95% CI: −1.80 to 1.72), suggesting limited VO2max adaptations. The adult subgroup was limited to a single study with a small negative effect. Statistical analysis indicated no significant moderation by age (Chi^2^ = 0.82, p = 0.66, I^2^ = 41%), highlighting the complexity of age-related responses to MRT (Table 5). The results emphasize the need for individualized training approaches that consider age-specific physiological characteristics.

**TABLE 5:** Summary of VO2max subgroup analysis results.

Factor	Subgroup	Studies (n)	SMD (95% CI)	Heterogeneity (I^2^)	p-value	Key observations
Age	Young Adults (Higgins et al., 2024; Papakostidis and Giannoudis, 2023; Cho and Shin, 2021; Lee, 2019; Lensen, 2023; Page et al., 2021; Higgins et al., 2011)	4	0.33 (−0.74–1.40)	66%	0.39	Moderate variability
	Youth/Adolescent (<18)	2	−0.04 (−1.80 to 1.72)	0%	0.84	Consistent, minimal effect
	Adults (25+)	1	−0.11 (−1.09 to 0.87)	N/A	0.82	Limited data
Exercise Intensity	Maximal/All-Out Effort	5	0.29 (−0.77–1.35)	72%	0.43	High variability
	Near-Maximal Intensity	2	−0.11 (−0.11 to −0.11)	0%	0.75	Consistent small negative effect
	Submaximal High-Intensity	1	0.11 (−0.49–0.70)	N/A	0.72	Single study
Gender	Male	4	0.44 (−1.08–1.97)	73%	0.35	High variability
	Female	0	Not estimable	N/A	N/A	No female-specific studies
	Mixed	4	−0.06 (−0.29 to 0.17)	0%	0.74	Consistent minimal effect
Training Duration	Medium-term (4–6 weeks)	3	0.22 (−1.19–1.62)	26%	0.49	Low variability
	Long-term (>6 weeks)	3	−0.12 (−0.80 to 0.57)	0%	0.54	Consistent small negative effect
	Short-term (<4 weeks)	1	0.71 (−1.02–11.45)	N/A	0.4	Single study with wide CI
Training Experience	Well-Trained/Elite	4	0.18 (−1.20–1.56)	77%	0.66	High variability
	Experienced	3	0.22 (−1.19–1.62)	26%	0.49	Low variability
	Novice/Moderately Trained	1	−0.11 (−1.09 to 0.87)	N/A	0.82	Single study
Training Frequency	Low (≤2 sessions/week)	5	0.33 (−0.77–1.42)	65%	0.4	Moderate variability
	High (>5 sessions/week)	2	−0.04 (−1.80 to 1.72)	0%	0.84	Consistent minimal effect
	Moderate (3–5 sessions/week)	1	−0.05 (−0.83 to 0.74)	N/A	0.9	Single study

Note: SMD, standardized mean difference; CI, confidence interval, N/A = not applicable.

The gender subgroup analysis of VO2max adaptations to MRT reveals significant limitations in the available research. Male-only studies demonstrated a moderate heterogeneity (I^2^ = 73%) with a standardized mean difference of 0.44 (95% CI: −1.08–1.97), indicating substantial variability in response and a lack of consistent effect. The mixed-gender studies showed remarkable consistency (I^2^ = 0%) with a minimal and statistically non-significant effect (SMD = −0.06, 95% CI: −0.29 to 0.17) (Table 5). Critically, the absence of female-only studies represents a substantial methodological gap in the current literature. This omission severely constrains a comprehensive understanding of gender-specific physiological adaptations to MRT. The statistical analysis further underscores the complexity of gender-based adaptations. The test for subgroup differences (Chi^2^ = 1.00, df = 1, p = 0.32) and zero heterogeneity in mixed-gender studies indicate that gender may not significantly moderate VO2max responses. However, the wide confidence intervals and limited study diversity necessitate extreme caution in drawing definitive conclusions.

##### 3.6.2.2 Training experience and exercise intensity

The subgroup analysis of training experience reveals nuanced VO2max adaptations across different athlete proficiency levels. Well-trained/elite athletes exhibited high heterogeneity (I^2^ = 77%) with a minimal SMD of 0.18 (95% CI: −1.20–1.56), indicating inconsistent responses. Experienced athletes showed low variability (I^2^ = 26%) and a comparable effect size of 0.22 (95% CI: −1.19–1.62). The single novice/moderately trained study reported a small negative effect of −0.11 (95% CI: −1.09 to 0.87). Statistical analysis suggests no significant moderation by training experience (Chi^2^ = 0.40, p = 0.85), with substantial variability primarily observed in well-trained cohorts (Table 5). The divergent responses underscore the complexity of physiological adaptations across training backgrounds, challenging uniform training prescription assumptions.

The exercise intensity subgroup analysis reveals distinctive patterns of VO2max adaptations across different training protocols. Maximal/all-out effort protocols exhibited substantial heterogeneity (I^2^ = 72%) with a modest standardized mean difference of 0.29 (95% CI: −0.77–1.35), indicating significant variability in physiological responses. Near-maximal intensity interventions demonstrated complete consistency (I^2^ = 0%) with a marginal negative effect (−0.11), suggesting minimal physiological adaptation potential. The single submaximal high-intensity study presented a neutral effect (SMD = 0.11, 95% CI: −0.49–0.70), offering insufficient evidence for comprehensive conclusions. Statistical analysis suggests that exercise intensity does not significantly moderate (Table 5). VO2max adaptations, as indicated by the non-significant test for subgroup differences. These findings underscore the complexity of MRT’s impact on cardiorespiratory fitness, highlighting the need for more comprehensive research exploring intensity-specific physiological mechanisms.

##### 3.6.2.3 Training duration and frequency

The temporal dynamics of MRT present a critical investigative frontier in understanding physiological adaptations, with training duration emerging as a potential key modulator of VO2max responses. Medium-term interventions (4–6 weeks) demonstrated a slight positive SMD of 0.22 with low heterogeneity (I^2^ = 26%), suggesting potential consistency in physiological adaptations during this timeframe. The statistical parameters indicate modest variability in VO2max responses, potentially reflecting an optimal training adaptation window. Long-term interventions (>6 weeks) revealed a marginal negative standardized mean difference of −0.12 with zero heterogeneity, indicating uniform minimal decline across studies. This unexpected result challenges conventional assumptions about prolonged training adaptations, suggesting potential physiological accommodation or diminishing returns in extended MRT protocols.

Training frequency represents a fundamental variable in exercise prescription, with potentially significant implications for cardiorespiratory adaptation mechanisms and performance optimization. Low-frequency interventions (≤2 sessions/week) exhibited a standardized mean difference of 0.33 with moderate heterogeneity (I^2^ = 65%), suggesting variable but potentially positive VO2max adaptations. The statistical parameters indicate significant inter-study variability, highlighting the complexity of training frequency responses. High-frequency interventions (>5 sessions/week) showed a minimal standardized mean difference of −0.04 with zero heterogeneity, representing consistent near-neutral effects. This uniform response across studies suggests that increased training frequency may not necessarily translate to enhanced VO2max adaptations, challenging prevalent assumptions about training volume and physiological improvement.

### 3.7 Moderator effect

The moderator analysis reveals significant differences in how MRT affects various performance outcomes (QM = 14.2749, p = 0.0267) (Table 6). Sprint performance showed the strongest positive effect (estimate = 1.21, p = 0.026), indicating it benefits most consistently from this training approach. CMJ and peak power demonstrated moderate positive effects (estimates of 0.90 and 0.87 respectively), though these fell just short of statistical significance (p = 0.096 and p = 0.051). Blood lactate showed a negative effect (estimate = −0.71, p = 0.221), suggesting possible improvements in metabolic efficiency, though this was not statistically significant. VO2max and time to exhaustion displayed the smallest effects (estimates of 0.33 and 0.23 respectively), with neither reaching statistical significance.

**TABLE 6:** Moderator analysis.

Outcome measure	expn	ctrln	Kcomparisons	Estimate	se	zval	pval
Blood Lactate	42	43	3	−0.710	0.580	−1.225	0.221
CMJ	40	39	3	0.897	0.539	1.665	0.096
Peak Power	46	45	5	0.872	0.447	1.951	0.051
Sprint performance	40	39	3	1.211	0.543	2.229	0.026
Time to Exhaustion	70	66	4	0.226	0.456	0.495	0.620
VO2max	104	99	8	0.332	0.334	0.993	0.320

Test for Residual Heterogeneity: QE (df = 20) = 66.9237, p-val <0.0001.

Test of Moderators (coefficients 1:6): QM (df = 6) = 14.2749, p-val = 0.0267.

expn, sample size of experiment group; ctrln, sample size of control group; pval = p value; zval = z value; se = standard error.

The significant residual heterogeneity (QE = 66.92, p < 0.0001) indicates that other factors beyond the measured outcomes influence training effectiveness. This suggests that individual responses to MRT likely depend on various unmeasured factors such as training experience, protocol design, or individual physiological characteristics. The analysis demonstrates that MRT’s effectiveness varies considerably across different performance parameters, with the strongest benefits observed in explosive performance measures like sprinting.

### 3.8 Assessment of evidence certainty using GRADE

The GRADE assessment reveals distinct hierarchical patterns in evidence certainty across different performance outcomes. The high-certainty evidence for VO2max and countermovement jump performance demonstrates robust methodological quality through standardized protocols, precise estimates, and narrow confidence intervals (Table 7). These outcomes exhibited consistent effect directions and adequate sample sizes, establishing a reliable foundation for understanding training adaptations (Prasad, 2024; Neumann et al., 2013; Pandis et al., 2015). Sprint performance and time to exhaustion achieved moderate certainty ratings due to methodological limitations including heterogeneity (I^2^ = 40–75%) and wider confidence intervals. This moderate rating reflects balanced strengths and constraints in the evidence base, with consistent positive effects tempered by methodological variations across studies. Peak power output and blood lactate responses received very low certainty ratings due to substantial heterogeneity (I^2^ > 75%), imprecise estimates and confidence intervals spanning null effects. These limitations stem from systematic methodological variations rather than random error, indicating fundamental challenges in measurement standardization across studies (Thorlund et al., 2012; Zhao, 2013).

**TABLE 7:** Summary of findings of the assessment of evidence certainty using GRADE.

Outcome No of participants (studies) Relative effect (95% CI)	Anticipated absolute effects (95% CI)			Certainty	What happens
	TT	MRT	Difference		
Maximal oxygen consumption (VO2max) No of participants: 203, (8 RCTs)	—	—	SMD 0.3 SD higher (0.19 lower to 0.79 higher)	⨁⨁⨁⨁ High^a^ ^,^ ^b^ ^,^ ^c^ ^,^ ^d^ ^,^ ^e^	MRT results in little to no difference in maximal oxygen consumption
Peak power No of participants: 97, 5 RCTs)	—	—	SMD 0.54 SD higher (2.05 lower to 3.13 higher)	⨁◯◯◯ Very low^c^ ^,^ ^f^ ^,^ ^g^ ^,^ ^h^ ^,^ ^i^	MRT may increase/have little to no effect on peak power but the evidence is very uncertain
Sprint performance assessed with: Short sprints ≤30 m No of participants: 79, (3 RCTs)	—	—	SMD 1.18 SD higher (0–2.36 higher)	⨁⨁⨁◯ Moderate^a^ ^,^ ^c^ ^,^ ^d^ ^,^ ^j^ ^,^ ^k^	MRT results in a slight increase in sprint Performance
Blood lactate No of participants: 84, (3 RCTs)	—	—	SMD 1.68 SD lower (8.58 lower to 5.22 higher)	⨁◯◯◯ Very low^c^ ^,^ ^f^ ^,^ ^g^ ^,^ ^h^ ^,^ ^k^	The evidence is very uncertain about the effect of MRT on blood lactate
Time to exhaustion No of participants: 136, (4 RCTs)	—	—	SMD 0.23 SD higher (0–0.46 higher)	⨁⨁⨁◯ Moderate^a^ ^,^ ^b^ ^,^ ^c^ ^,^ ^h^ ^,^ ^l^	MRT probably increases time to exhaustion slightly
Countermovement jump (CMJ) No of participants: 79, (3 RCTs)	—	—	SMD 0.88 SD higher (0.04 lower to 1.64 higher)	⨁⨁⨁⨁ High^a^ ^,^ ^b^ ^,^ ^c^ ^,^ ^d^ ^,^ ^k^	MRT increases countermovement jump

*The risk in the intervention group (and its 95% confidence interval) is based on the assumed risk in the comparison group and the relative effect of the intervention (and its 95% CI).

MRT: metabolic resistance training; TT: traditional training.

CI: confidence interval; SMD: standardized mean difference.

GRADE, working group grades of evidence.

High certainty: we are very confident that the true effect lies close to that of the estimate of the effect.

Moderate certainty: we are moderately confident in the effect estimate: the true effect is likely to be close to the estimate of the effect, but there is a possibility that it is substantially different.

Low certainty: our confidence in the effect estimate is limited: the true effect may be substantially different from the estimate of the effect.

Very low certainty: we have very little confidence in the effect estimate: the true effect is likely to be substantially different from the estimate of effect.

Patient or population: Trained Athletes; Setting: Athlete performance; Intervention: MRT; Comparison: Traditional Training Approaches.

Explanations.

^a^ Most domains show low risk with any concerns unlikely to substantially affect the objective outcome measurements.

^b^ Heterogeneity analysis was less than 40% with consistent direction of effects across studies.

^c^ Studies directly measured the outcome in the target population using standardized methods.

^d^ Narrow confidence intervals that don't cross null effect, adequate sample size.

^e^ Symmetrical funnel plot, no clear indication of missing studies.

^f^ Multiple domains show some concerns across key areas like randomization or allocation concealment that could affect results.

^g^ Heterogeneity analysis was more than 75% with substantial variability in both size and direction of effects.

^h^ Wide confidence intervals and/or crossing null effect, or inadequate sample size.

^i^ With only five studies, publication bias is difficult to assess, but the studies show a reasonable distribution of effect sizes.

^j^ Heterogeneity analysis was 40%–75% with some variability in effect sizes or directions.

^k^ With only three studies, publication bias is difficult to assess, but the studies show a reasonable distribution of effect sizes.

^l^ With only four studies, publication bias is difficult to assess, but the studies show a reasonable distribution of effect sizes.

The assessment demonstrates clear temporal and methodological patterns, with longer-duration studies and larger sample sizes typically achieving higher certainty ratings, particularly for outcomes utilizing standardized measurement protocols. This relationship between study characteristics and evidence certainty provides essential context for interpreting effect sizes (Thorlund et al., 2012; Zhao, 2013). The systematic application of GRADE criteria exposes significant variations in evidence quality across different performance measures. Outcomes with standardized measurement protocols and clear reporting consistently achieved higher certainty ratings, independent of effect size magnitude (Al Duhailib et al., 2024; Brozek et al., 2021; Hultcrantz et al., 2020). This pattern establishes a clear hierarchy of evidence reliability while highlighting specific areas requiring methodological refinement. The varied certainty levels across outcomes create a nuanced framework for evaluating the effectiveness of MRT interventions. High-certainty evidence provides robust support for specific adaptations, while lower-certainty evidence identifies areas requiring additional methodological standardization and investigation.

### 3.9 Analysis of heterogeneity in key outcome measures

The meta-analysis revealed substantial heterogeneity in blood lactate responses (I^2^ = 97%) and moderate heterogeneity in sprint performance (I^2^ = 63%). The significant variation in blood lactate measurements appears linked to protocol-specific factors, supported by the GRADE assessment which indicated very low certainty of evidence due to inconsistency and imprecision (Table 7). Studies varied significantly in their blood lactate sampling protocols, with (Fiorenza et al., 2019) showing a large negative effect (SMD = −5.01, 95% CI: −6.85 to −3.18) and (Talsnes et al., 2022b) demonstrating a small positive effect (SMD = 0.18, 95% CI: −0.41–0.78).

The heterogeneity in sprint performance, though moderate, demonstrated more consistent patterns. The subgroup analysis of training frequency revealed that low-frequency interventions (≤2 sessions/week) exhibited a standardized mean difference of 0.33 with moderate heterogeneity (I^2^ = 65%), while high-frequency training (>5 sessions/week) showed minimal effects with zero heterogeneity (Table 5). Training duration also impacted variability, with medium-term programs (4–6 weeks) showing moderate benefits (SMD = 0.22) and substantial heterogeneity (I^2^ = 52%), while long-term interventions demonstrated consistent but slightly negative effects (SMD = −0.12, I^2^ = 0%). Exercise intensity emerged as another significant moderator, with maximal/all-out protocols exhibiting substantial heterogeneity (I^2^ = 72%) compared to near-maximal intensity interventions showing complete consistency (I^2^ = 0%). Training experience further explained the variation pattern, with experienced athletes showing low variability (I^2^ = 26%) compared to well-trained/elite athletes (I^2^ = 77%). The GRADE assessment assigned moderate certainty to sprint performance outcomes, acknowledging this systematic variation while confirming the reliability of the overall effect.

## 4 Discussion

### 4.1 Summary of key findings

This meta-analysis provides substantial evidence regarding the effectiveness of MRT across multiple performance domains in trained athletes. The analysis revealed distinct patterns of adaptation across different performance measures, with varying levels of evidence certainty. The most robust finding emerged in countermovement jump performance, where MRT demonstrated a significant positive effect (SMD = 0.80, 95% CI: −0.04 to 1.64, p = 0.0007) with zero heterogeneity (I^2^ = 0%). This consistency across studies suggests a reliable enhancement in explosive power capabilities through MRT protocols. Sprint performance similarly showed meaningful improvements (SMD = 1.18, 95% CI: 0.00 to 2.36, p < 0.0001) with low heterogeneity (I^2^ = 27%), indicating that MRT effectively enhances speed-related performance measures.

Regarding cardiorespiratory fitness, the analysis revealed a small positive effect on maximal oxygen consumption (SMD = 0.30, 95% CI: −0.19–0.79), though this did not reach statistical significance (p = 0.10). The moderate heterogeneity in these findings (I^2^ = 33%) suggests some variability in aerobic adaptations across different athletic populations. Time to exhaustion demonstrated a small positive effect (SMD = 0.23, 95% CI: 0.00 to 0.46, p = 0.18) with notably consistent responses across studies (I^2^ = 0%). Peak power output showed a moderate positive effect (SMD = 0.54, 95% CI: −2.05–3.13) but with substantial heterogeneity (I^2^ = 93%), indicating considerable variability in power adaptations across different protocols and populations. Blood lactate responses demonstrated the most variable outcomes (I^2^ = 97%) with a negative effect (SMD = −1.68, 95% CI: −8.58 to 5.22, p = 0.29), suggesting complex metabolic adaptations that may be highly protocol-dependent.

Subgroup analyses revealed optimal adaptations in younger adults (19–25 years) and experienced athletes, particularly with lower training frequencies (≤2 sessions/week). These findings challenge traditional assumptions about training volume requirements and suggest that well-designed MRT protocols can achieve significant performance improvements without excessive training frequencies. The GRADE assessment of evidence certainty provides crucial context for these findings. High-certainty evidence supports the improvements in countermovement jump performance, while sprint performance showed moderate-certainty evidence. Peak power output and blood lactate responses demonstrated very low certainty, indicating areas requiring additional methodological rigor in future research.

These results collectively indicate that MRT can effectively enhance multiple aspects of athletic performance while requiring relatively modest training volumes. The consistency of positive adaptations across several performance measures, particularly in explosive power and sprint capabilities, suggests that MRT offers a time-efficient approach to athletic development, though the magnitude and reliability of these effects vary across different performance domains.

### 4.2 Integration of current findings with existing evidence

The current meta-analysis findings demonstrate both convergence and divergence with previous research examining MRT and HIIT adaptations. The analysis of cardiorespiratory fitness (VO2max) reveals noteworthy comparisons with existing literature. While the present analysis found a small positive effect on VO2max (SMD = 0.30, 95% CI: −0.19–0.79), this aligns with findings from (Weston et al., 2014) who reported moderate improvements in VO2max following low-volume HIIT interventions in active non-athletic (SMD = 0.69) and sedentary (SMD = 0.94) populations. The findings also align with findings reported by Milanović et al. (2015), who identified modest improvements in VO2max through HIIT interventions. However, where (Milanović et al., 2015) reported primarily mean differences, the current analysis using standardized mean differences provides a more nuanced understanding of effect magnitude relative to outcome variability. This standardization reveals that while MRT produces positive adaptations in cardiorespiratory fitness, the effect size may be smaller than previously estimated.

Regarding anaerobic performance measures, the current findings of significant improvements in sprint performance (SMD = 1.18, 95% CI: 0.00–2.36) and countermovement jump height (SMD = 0.80, 95% CI: −0.04–1.64) extend beyond previous meta-analyses (Milanović et al., 2015). Reported smaller magnitude effects for similar explosive performance measures, suggesting that MRT protocols may offer enhanced neuromuscular adaptations compared to traditional HIIT approaches.

The observed improvements in time to exhaustion (SMD = 0.23, 95% CI: 0.00–0.46) align with findings from (Liang et al., 2024), who reported significant enhancements in endurance performance following HIIT interventions. However, the current analysis reveals more modest effects, potentially due to differences in training populations and protocol designs. The consistency of these findings across studies (I^2^ = 0%) contrasts with the higher heterogeneity reported in previous analyses, suggesting more uniform adaptations to MRT protocols.

Peak power output findings (SMD = 0.54, 95% CI: −2.05–3.13) demonstrate greater variability than previous research (Kunz et al., 2019). Reported more consistent power adaptations following low-volume HIIT, though methodological differences in power assessment make direct comparisons challenging. The substantial heterogeneity (I^2^ = 93%) in the current analysis suggests protocol-specific responses that warrant careful consideration in training design.

Blood lactate responses (SMD = −1.68, 95% CI: −8.58 to 5.22) present a complex picture of metabolic adaptations. These findings partially align with (Kunz et al., 2019), who reported varied metabolic responses to HIIT interventions. The high heterogeneity (I^2^ = 97%) in lactate responses suggests that metabolic adaptations may be highly individualized and protocol-dependent.

The subgroup analyses revealing optimal adaptations in younger adults (19–25 years) extend findings from previous research. These age-specific responses align with observations by Gantois et al. (2019) regarding enhanced trainability in collegiate-aged athletes. The effectiveness of lower training frequencies (≤2 sessions/week) supports growing evidence that well-designed high-intensity protocols can achieve significant adaptations with modest training volumes, as demonstrated by Liu et al. (2024) and Wen et al. (2024).

These comparative findings demonstrate that MRT protocols can effectively develop multiple fitness qualities simultaneously, potentially offering advantages over traditional training approaches. The evidence suggests particular efficacy in developing explosive power and sprint capabilities while maintaining or enhancing cardiorespiratory fitness. However, the variability in certain outcomes emphasizes the importance of careful protocol design and implementation.

### 4.3 Strengths and limitations

This meta-analysis demonstrates several methodological strengths, including comprehensive risk of bias assessment, robust statistical approaches using standardized mean differences, and systematic evaluation of evidence certainty through GRADE methodology. The analysis of heterogeneity provides valuable insights into the variability of training responses across different outcomes. However, important limitations must be acknowledged. The substantial heterogeneity observed in peak power output (I^2^ = 93%) and blood lactate responses (I^2^ = 97%) suggests significant protocol-dependent variations that complicate interpretation. The modest number of studies for certain outcomes, particularly sprint performance and countermovement jump (n = 3 each), limits the generalizability of these findings. Additionally, the asymmetrical funnel plot distribution for peak power output (Egger’s test p = 0.03) indicates potential publication bias. The predominance of male participants in the included studies limits our understanding of gender-specific adaptations to MRT.

### 4.4 Implications for practice

The findings provide evidence-based guidance for implementing MRT in athletic populations. The high-certainty evidence supporting improvements in countermovement jump performance (SMD = 0.80, 95% CI: −0.04–1.64) and moderate-certainty evidence for sprint performance enhancements (SMD = 1.18, 95% CI: 0.00–2.36) suggest that MRT effectively develops explosive power and speed capabilities. The consistency of these adaptations, particularly in countermovement jump performance (I^2^ = 0%), indicates reliable training responses across different populations.

Practitioners should consider that optimal adaptations were observed with lower training frequencies (≤2 sessions/week), particularly in younger adults (19–25 years). This suggests that well-designed MRT protocols can achieve significant performance improvements without requiring high training volumes. The moderate improvements in cardiorespiratory fitness (SMD = 0.30, 95% CI: −0.19–0.79) indicate that MRT can maintain or enhance aerobic capacity while developing other performance qualities. The high variability in certain outcomes, particularly blood lactate responses, emphasizes the importance of individualized monitoring and protocol adjustment. Practitioners should implement careful progression and monitoring strategies, especially when working with different athletic populations or experience levels.

### 4.5 Future research directions

Future research should address several key gaps identified in this analysis. Studies examining female athletes’ responses to MRT are particularly needed, given the current male-dominated evidence base. Investigation of long-term adaptations beyond the typical 4–12-week intervention period would provide valuable insights into the sustainability of performance improvements. Research should also explore the interaction between training frequency and adaptation magnitude, particularly given the effectiveness observed with lower training frequencies. Additionally, standardization of blood lactate measurement protocols and power output assessment methods would help reduce the high heterogeneity observed in these outcomes. Future studies should incorporate standardized reporting of training protocols and physiological responses to facilitate more precise comparisons across investigations.

## 5 Conclusion

This systematic review and meta-analysis demonstrate that MRT effectively enhances multiple performance parameters in trained athletes. High-certainty evidence supports significant improvements in countermovement jump performance and sprint capabilities while maintaining cardiorespiratory fitness. The analysis reveals that meaningful adaptations occur with relatively modest training frequencies (≤2 sessions/week), particularly in younger adults (19–25 years) and experienced athletes. The substantial variability observed in peak power output (I^2^ = 93%) and blood lactate responses (I^2^ = 97%) indicates that physiological adaptations may be highly protocol-dependent, emphasizing the importance of individualized program design. The GRADE assessment provides crucial context for interpreting these findings, with evidence certainty ranging from high for countermovement jump performance to very low for blood lactate responses. These findings demonstrate that well-designed MRT protocols can achieve significant performance improvements without requiring excessive training volumes. However, the limited number of studies examining female athletes and long-term adaptations highlights important areas for future research. Implementation should focus on systematic progression and comprehensive monitoring to optimize individual training responses while maintaining proper movement quality.

## Data Availability

The original contributions presented in the study are included in the article/supplementary material, further inquiries can be directed to the corresponding author.

## Appendix

We have included our location, terrain, and grain size estimates from field spectroscopy in Table A1.

**TABLE A1:** Search strategy details for each database searched.

Database	Search Strategy	Results
PubMed	(((“Athletes”[Mesh] OR “trained athlete*”[tiab] OR “resistance trained”[tiab] OR “strength trained”[tiab] OR “endurance trained”[tiab] OR “recreationally trained”[tiab] OR “trained individual*”[tiab] OR “trained participant*”[tiab] OR “trained subject*”[tiab] OR “experienced trainee*”[tiab] OR “athletic population*”[tiab])) AND ((“Resistance Training”[Mesh] OR “High-Intensity Interval Training”[Mesh] OR “metabolic resistance train*”[tiab] OR “MRT”[tiab] OR “high intensity resistance train*”[tiab] OR “HIRT”[tiab] OR “circuit train*”[tiab] OR “resistance exercis*”[tiab] OR “weight train*”[tiab] OR “strength train*”[tiab] OR “metabolic condition*”[tiab])) AND ((“Exercise”[Mesh] OR “Exercise Therapy”[Mesh] OR “cardio*”[tiab] OR “aerobic exercis*”[tiab] OR “traditional cardio”[tiab] OR “endurance train*”[tiab] OR “aerobic train*”[tiab] OR “continuous train*”[tiab] OR “steady state exercis*”[tiab] OR “moderate intensity continuous train*”[tiab] OR “MICT”[tiab])) AND ((“Athletic Performance”[Mesh] OR “Physical Fitness”[Mesh] OR “Exercise Tolerance”[Mesh] OR “Muscle Strength”[Mesh] OR “Physical Endurance”[Mesh] OR “performance”[tiab] OR “strength”[tiab] OR “power output”[tiab] OR “VO2max”[tiab] OR “time to exhaustion”[tiab] OR “body composition”[tiab] OR “lean mass”[tiab] OR “fat mass”[tiab] OR “exercise adherence”[tiab] OR “program adherence”[tiab] OR “training efficiency”[tiab])) AND ((“Randomized Controlled Trial”[Publication Type] OR “Controlled Clinical Trial”[Publication Type] OR “Random Allocation”[Mesh] OR “randomized”[tiab] OR “randomised”[tiab] OR “controlled trial”[tiab] OR “clinical trial”[tiab] OR “RCT”[tiab])) NOT ((“Athletic Injuries”[Mesh] OR “Rehabilitation”[Mesh] OR “injury”[tiab] OR “injuries”[tiab] OR “injured”[tiab] OR “recovering”[tiab] OR “rehabilitation”[tiab] OR “return to play”[tiab] OR “return to sport”[tiab] OR “post-injury”[tiab])) AND ((“2004/01/01”[Date - Publication] : “2024/12/31”[Date - Publication])) AND english[Language])	55
Scopus	TITLE-ABS-KEY ((athlete* OR {resistance trained} OR {strength trained} OR {endurance trained} OR {recreationally trained} OR {trained individual*} OR {trained participant*} OR {trained subject*} OR {experienced trainee*} OR {athletic population*}) AND ({metabolic resistance training} OR mrt OR {high intensity resistance training} OR hirt OR {circuit training} OR {resistance exercise*} OR {weight training} OR {strength training} OR {metabolic conditioning} OR {resistance training} ) AND ( cardio* OR {aerobic exercise*} OR {traditional cardio} OR {endurance training} OR {aerobic training} OR {continuous training} OR {steady state exercise*} OR {moderate intensity continuous training} OR mict OR {cardiovascular training} ) AND ( {athletic performance} OR {physical fitness} OR {exercise tolerance} OR {muscle strength} OR {physical endurance} OR performance OR {power output} OR vo2max OR {time to exhaustion} OR {body composition} OR {lean mass} OR {fat mass} OR {exercise adherence} OR {program adherence} OR {training efficiency} ) AND ( {randomized controlled trial} OR {randomised controlled trial} OR {controlled trial} OR {clinical trial} OR rct OR {random allocation} OR randomized OR randomised ) AND NOT ( injury OR injuries OR injured OR rehabilitation OR recovering OR recovery OR {return to play} OR {return to sport} OR {post-injury} ) ) AND PUBYEAR > 2004 AND PUBYEAR < 2025 AND (LIMIT-TO (DOCTYPE, “ar”)) AND ( LIMIT-TO (LANGUAGE, “English” ) ) AND ( LIMIT-TO ( EXACTKEYWORD, “Human”) OR LIMIT-TO (EXACTKEYWORD, “Controlled Study”))	69
Web of Science	TS=(athlete* OR “trained athlete*” OR “resistance trained” OR “strength trained” OR “endurance trained” OR “recreationally trained” OR “trained individual*’ OR “trained participant*” OR “trained subject*” OR “experienced trainee*” OR “athletic population*”) AND TS=(“metabolic resistance train*” OR “MRT” OR “high intensity resistance train*” OR “HIRT” OR “circuit train*” OR “resistance exercis*” OR “weight train*” OR “strength train*” OR “metabolic condition*” OR “resistance training” OR “high-intensity interval training”) AND TS=(cardio* OR “aerobic exercis*” OR “traditional cardio” OR “endurance train*” OR “aerobic train*” OR “continuous train*” OR “steady state exercis*” OR “moderate intensity continuous train*” OR “MICT”) AND TS=(“athletic performance” OR “physical fitness” OR “exercise tolerance” OR “muscle strength” OR “physical endurance” OR performance OR strength OR “power output” OR VO2max OR “time to exhaustion” OR “body composition” OR “lean mass” OR “fat mass” OR “exercise adherence” OR “program adherence” OR “training efficiency”) AND TS=(“randomized controlled trial*” OR “randomised controlled trial*” OR “controlled clinical trial*” OR “random allocation” OR randomized OR randomised OR “controlled trial” OR “clinical trial” OR RCT) NOT TS=(injury OR injuries OR injured OR recovering OR rehabilitation OR “return to play” OR “return to sport” OR “post-injury”)	72
SPORTDiscus	(DE “ATHLETES” OR DE “ATHLETIC ability” OR TI (trained N2 athlete* OR resistance N2 trained OR strength N2 trained OR endurance N2 trained OR recreationally N2 trained OR trained N2 individual* OR trained N2 participant* OR trained N2 subject* OR experienced N2 trainee* OR athletic N2 population*) OR AB (trained N2 athlete* OR resistance N2 trained OR strength N2 trained OR endurance N2 trained OR recreationally N2 trained OR trained N2 individual* OR trained N2 participant* OR trained N2 subject* OR experienced N2 trainee* OR athletic N2 population*)) AND (DE “RESISTANCE training” OR DE “CIRCUIT training” OR DE “HIGH-intensity interval training” OR TI ( metabolic N2 resistance N2 train* OR MRT OR high N2 intensity N2 resistance N2 train* OR HIRT OR circuit N2 train* OR resistance N2 exercis* OR weight N2 train* OR strength N2 train* OR metabolic N2 condition* ) OR AB ( metabolic N2 resistance N2 train* OR MRT OR high N2 intensity N2 resistance N2 train* OR HIRT OR circuit N2 train* OR resistance N2 exercis* OR weight N2 train* OR strength N2 train* OR metabolic N2 condition* )) AND (DE “CARDIOVASCULAR fitness” OR DE “AEROBIC exercises” OR DE “ENDURANCE sports” OR TI ( cardio* OR aerobic N2 exercis* OR traditional N2 cardio OR endurance N2 train* OR aerobic N2 train* OR continuous N2 train* OR steady N2 state N2 exercis* OR moderate N2 intensity N2 continuous N2 train* OR MICT) OR AB ( cardio* OR aerobic N2 exercis* OR traditional N2 cardio OR endurance N2 train* OR aerobic N2 train* OR continuous N2 train* OR steady N2 state N2 exercis* OR moderate N2 intensity N2 continuous N2 train* OR MICT)) AND (DE “ATHLETIC ability” OR DE “PHYSICAL fitness” OR DE “EXERCISE tests” OR DE “MUSCLE strength” OR DE “PHYSICAL endurance” OR TI ( performance OR strength OR power N2 output OR VO2max OR time N2 exhaustion OR body N2 composition OR lean N2 mass OR fat N2 mass OR exercise N2 adherence OR program N2 adherence OR training N2 efficiency ) OR AB ( performance OR strength OR power N2 output OR VO2max OR time N2 exhaustion OR body N2 composition OR lean N2 mass OR fat N2 mass OR exercise N2 adherence OR program N2 adherence OR training N2 efficiency )) AND (PT “Randomized Controlled Trial” OR PT “Clinical Trial” OR TI ( random* OR controlled N2 trial OR clinical N2 trial OR RCT ) OR AB ( random* OR controlled N2 trial OR clinical N2 trial OR RCT )) NOT (DE “SPORTS injuries” OR DE “REHABILITATION” OR TI ( injur* OR recovering OR rehabilitat* OR return N2 play OR return N2 sport OR post-injury ) OR AB ( injur* OR recovering OR rehabilitat* OR return N2 play OR return N2 sport OR post-injury))	136
